# Bipolar Affective Disorder and Parkinson's Disease

**DOI:** 10.1155/2011/154165

**Published:** 2011-11-28

**Authors:** Birk Engmann

**Affiliations:** Department of Neurology, Fachklinikum Brandis, Am Wald, 04821 Brandis, Germany

## Abstract

Little is known about comorbidities of bipolar disorder such as Parkinson's disease. A case history and a literature survey indicate that bipolar disorder is linked with or influences Parkinson's disease and vice versa. Underlying mechanisms are poorly understood, and, more importantly, no treatment options are established in such double diagnoses. The few data in comorbid Parkinson cases seem to point to a rapid cycling pattern of bipolar symptoms. With regard to therapeutic intervention, the literature supports pramipexole for treatment of both Parkinson and depressive symptoms in bipolar depression. Lithium, the mood stabilizer of choice for treating manic states, is problematical for use in Parkinson patients because of its side effects. Valproate might be an alternative, especially for treatment of rapid cycling.

## 1. Introduction

In some cases, bipolar disorder is linked with Parkinson's disease. Little is known of such comorbidities, and no treatment options have been recommended for such exceptional cases. For this paper, a MEDLINE literature search was used. Terms used for the search were bipolar disorder, bipolar depression, mania, and Parkinson's disease.

## 2. Case History

A 69-year-old man was admitted to hospital as having a depressive episode in Parkinson's disease. According to his wife, even as a young man he had often and easily become very agitated. His wife had counted such behaviour as a personal trait and had ordered her life to deal with his phases of high activity and his irritated, angry moods. When he was in those states, which lasted a few weeks and appeared to her to occur randomly, he had lots of ideas but he had been able to act on them. This pattern of behaviour had lasted his whole life until the onset of Parkinson's disease at the age of 61 years. The first signs of his Parkinson's disease were a stiffness of movement of the right arm and a tremor in the right hand. With the beginning of Parkinson's disease, novel behaviours, such as having depressive states and avolition, occurred more often. In addition, the previous symptoms of hyperactivity and angry mood became stronger. There were also phases of hypersexuality. He also spent a lot of money. In addition, he engaged in strenuous work in his garden, despite the weakening of his motor abilities and physical resilience. He labored until he was physically exhausted. When he was cutting timber, his wife asked him to rest for a while, but after five minutes he resumed working, even though he felt weary. In his hyperactive state he was not open to reasonable arguments. In contrast, there were several days in which he was “normal” and yet other days when he felt gloomy and had no drive. His states of mood changed within a few days and over time worsened. Other symptoms which occurred with his Parkinson's disease were forgetfulness and loss of attention. The patient's report of his symptoms agrees with that of his wife. He added that usually his mood is better in the evening than in the morning. Such mood changes over a day or in the course of the year are not related to changes in motor function caused by Parkinson's disease. His Parkinson's disease had been treated with dopamine agonists (pramipexole, later switched to rotigotine) at the beginning and one year later with additional levodopa medication. Because of recent lively dreams accompanied by a difficulty in distinguishing dream content from reality, the antipsychotic quetiapine in low dose was administered and with success. Visual hallucinations occurred only once in his life. This was after a heart operation which he had undergone one year after onset of Parkinson's disease.

The patient's family history shows an abundance of affective disorders. His mother had both a lifelong bipolar disorder and Parkinson's disease in old age, just as the patient had. Other relatives of the patient also had affective disorders, but not so severe. The patient's brother had a depressive episode followed by a hypomanic state. Of the patient's two daughters, one suffered from recurrent depressions but no manic states. The other daughter is described as always overactive, having lots of ideas, but had never been in psychic therapy. Her symptoms did not appear to her to be serious enough to require therapy.

## 3. Does Dopaminergic Medicine Lead to the Development of Bipolar Disorder in Patients?

The history of the patient reveals cyclothymic behavioral traits. It is supposed that the onset of Parkinson's disease worsened those traits, so that his behavior switched from “normal” to exhibiting bipolar disorder. Frequency of switches of (hypo)mania and depression refers to “ultra-rapid cycling”.

In the literature, the influence of the dopamine system on bipolar disorder is discussed. Manic states are related to increased dopaminergic activity, whereas a depression is caused by the opposite effect. An experimental irritation of the substantia nigra induces manic behaviour [1]. It is also reported that manic behaviour appears in the on phases [2]. In that case history, the question arises whether the starting of Parkinson medication released bipolar symptoms. On the other hand, dopamine-2 receptor agonist pramipexole, which was first administered to the patient, is recommended for therapy-resistant bipolar and unipolar depression [3].

In general, dementia, sleep disturbances, sexual malfunctions, depressive episodes, dysthymia, anxiety, and panic attacks occur together with Parkinson's disease. Others report about the possibility of development of bipolar disorder especially in Parkinson patients: Kim et al. [4], for instance, described two such patients with no family history of affective disorders. Scappa et al. [5] reported on three patients with rapid cycling who always had Parkinson symptoms together with tardive dyskinesia at the beginning of a depressive phase. When depression switched to mania, these symptoms disappeared.

In a review of literature by Goodwin and Jamison, [6] many neurotransmitter systems, such as dopaminergic, noradrenergic, serotonergic, and others, are said to be involved in bipolar disorder but none of them alone is considered to play a fundamental role in the development of the disease. It is not clear how bipolar disorder and Parkinson's disease might influence each other, or whether they might have a common origin.

## 4. Is Parkinson's Disease a Risk for Rapid Cycling?

Some case histories indicate a bipolar disorder with a rapid cycling pattern. Rapid cycling is defined being more than four phases per year, whereas ultrarapid cycling (URC) and ultrarapid ultradian cycling (URUC) are defined by switches within a few days and within a few hours, respectively. Kummer et al. [7] described a sample of six Parkinson patients with bipolar disorder. In all cases manic or hypomanic episodes had already appeared before the onset of Parkinson's disease and, furthermore, the authors supposed that Parkinson's disease accelerates the frequency of episodes.

Indeed, Kirov et al. [8] postulated a prominent role of catechol-o-methyltransferase (COMT) in rapid cycling in the form of lower activity of a COMT allele and polymorphism of serotonin transporter gene. Could COMT be a linkage to Parkinson comorbidity? But the study had a limitation: all patients also suffered from a comorbid DiGeorge-syndrome. Others support polymorphism of serotonin transporter gene [9], whereas Papadimitriou et al. [10] argued that genetic studies have not convincingly shown a genetic determination. All in all, the question if coincidence of Parkinson's is a risk factor of rapid cycling has not been answered yet. More data is needed. 

## 5. Treatment Options for Comorbid Parkinson's and Bipolar Patients

For the special case—the combination of Parkinson's and bipolar disorder—no specific data for treatment options exist. But some remedies are known to act on both diseases, and so it is possible to extrapolate this knowledge to that special case. Most publications report on pramipexole. Also reboxetine, mirtazapine, and nortriptyline are effective in treatment of depression in Parkinson patients [11]. But in order to diminish the amount of medication and to avoid possible side effects of additional antidepressants—because it acts both on Parkinson symptoms and depression—pramipexole is seen as an alternative to antidepressant drugs in depressed Parkinson patients [12]. Best support of pramipexole in the literature for Parkinson patients with mood disorder is for bipolar depression [3].

Quetiapine is used as an alternative to clozapine in Parkinson patients with psychotic symptoms. Data also support quetiapine for acute therapy of bipolar depression [13] and as a mood stabilizer too [14]. But some studies warn of risks to develop extrapyramidal motor symptoms (EPS) under treatment with atypical neuroleptics. Gao et al. [15] pointed out that bipolar patients had a higher risk for EPS than patients with schizophrenia. In comorbid Parkinson-bipolar disorder patients these findings are very important, but sufficient data in comorbid Parkinson's bipolar disorder patients are needed to establish more precise treatment options in future. An alternative mood stabilizer in bipolar-II disorder is lamotrigine, but there are no data about administration in comorbidity patients. In bipolar-I patients lithium is the mood stabilizer of choice [14], but in contrast, lithium could worsen Parkinson symptoms and causes tremor and weakness especially. Despite of that, Kim et al. [4] pointed out good efficacy of lithium, especially when combined with clozapine, for manic phases in comorbid Parkinson's bipolar disorder patients. In conclusion, too little is known about treatment options in bipolar patients with comorbid disease. Further investigations are needed.
